# Cascades of quasi-bound states in the continuum

**DOI:** 10.1515/nanoph-2025-0238

**Published:** 2025-08-05

**Authors:** Nikolay Solodovchenko, Mikhail Bochkarev, Kirill Samusev, Mikhail Limonov

**Affiliations:** School of Physics and Engineering, ITMO University, St. Petersburg, Russia; 65071 Ioffe Institute, Saint-Petersburg, Russia

**Keywords:** bound states in the continuum, Fabry–Pérot resonances, Mie scattering, ring resonator, split ring resonator, Fano resonance

## Abstract

Future technologies aim to radically increase photonic integration, which can be achieved either by structuring the materials or by cleverly manipulating photonic resonances. The latter method involves several tunable resonant modes in a single simple structure. Here we demonstrate experimentally and theoretically the existence of multiple cascades of quasi-bound states in the continuum in single dielectric resonators with rectangular cross sections – in rings, split rings, and cuboids, which form the basis of modern photonics. The effect is determined by the photonic structure of such resonators: it consists of individual galleries, each starting with a transverse Fabry–Pérot-like resonance in height or width and continuing with an equidistant sequence of longitudinal modes. When only one of the transverse dimensions in the spectrum changes, only one gallery type is predominantly shifted, leading to the avoiding crossings with the other gallery and the formation of multiple cascades of quasi-bound states in the continuum via the Friedrich–Wintgen mechanism. This “Fabry–Pérot-tronic” has an obvious advantage over the “Mie-tronic”, whose only variable geometric parameter is the radius of the sphere. Such single dielectric resonators with cascades of quasi-bound states in the continuum can become building blocks for multichannel sensors, antennas, amplifiers, and lasers with a wide range of equidistant generation frequencies; in addition, such a simple resonator creates a new platform for multifrequency sensing using machine learning.

## 1 Introduction

Modern photonics relies on novel concepts and original ideas that define a new vector of development of practical applications. Mie-tronics [1], [2] is becoming a new paradigm in photonics, which is based on high-index dielectrics and is gradually replacing plasmonics with its insuperable problem of ohmic losses. A surge of attention to dielectric structures is associated with the observation of induced magnetic dipole resonances in subwavelength spherical nanoparticles [3], [4], [5], [6]. For the problem of light scattering by a sphere, Mie theory [7] can be used to calculate the resonance position, linewidth, and amplitude [8], [9], and each mode can be associated with a separate term in the Mie series. For modes within total internal reflection, the resonance linewidth can be extremely narrow with a position that depends on size and relative refractive index, and dielectric spheres can act as high-quality optical resonators [10]. Although initial studies of scattering by a sphere focused on the first multipoles, including magnetic dipole and electric quadrupole moments, later studies also discussed high-order multipoles. It is now clear that dielectric structures can lead to many new effects arising from the interference of several modes, such as the Kerker effect [11], Fano resonance [12], [13], optical anapoles [14], [15], and bound states in the continuum (BIC) [16], [17]. It should be noted that an ideal theoretical BIC with an infinite *Q* factor is unattainable in real objects of finite size [16]; in this case speaks of quasi-BIC (q-BIC) [18]. In this study we consider only q-BICs.

Mie resonances occur when the wavelength of light inside a spherical particle becomes comparable to its only geometric parameter, the diameter of the sphere, 2*R* ≈ *λ*/*n*, where *n* is the refractive index of the dielectric material, *R* is the radius of the sphere, and *λ* is the wavelength of light in a vacuum. Thus, the possibilities of manipulating photonic resonances due to the geometry of the sphere are limited to one variable. The situation changes radically when moving from a sphere to structures with sets of resonances, the eigenvalues of which can be changed practically independently. These are resonators of various shapes, having at least two pairs of parallel walls, like a rectangle or rhombus, which support Fabry–Pérot-like resonances. For definiteness, we will further call this field, by analogy with Mie-tronics, Fabry–Pérot-tronics. With this additional degree of freedom, Fabry–Pérot-tronics greatly enhances Mie-tronics’ ability to manipulate photonic resonances and observe impressive interference effects involving a large number of resonant modes.

Multimode interference effects have been observed in a dielectric cylinder [18] and ring with a rectangular cross section [17], [19]. In a cylindrical resonator, low-frequency resonances can be divided into two types of practically independent modes, Fabry–Pérot-like between the upper and lower walls and Mie-like in the plane of the cylinder cross section. Thus, by changing only the height of the cylinder, it is possible to achieve an interference region of two modes existing in one radiation channel, with the formation of q-BIC [18]. In a ring resonator, due to the presence of an internal side wall in a ring, it is possible to purposefully vary the coupling coefficient of two interfering modes and observe quadruplets of resonance states consisting of two exceptional points [20] connected by a Fermi arch [21] and two q-BICs, each adjacent to one of the exceptional points in the parametric space [22].

The key idea of Fabry–Pérot-tronics is based on the specific nature of the eigenmodes of rectangular dielectric resonators – ring, split ring, and cuboid. They can be represented as a finite waveguide with different boundary conditions at the ends. In the low-frequency region, the eigenmodes of a rectangular dielectric waveguide are conditionally divided into TE and TM mode resonances depending on the dominant component of the electric field. We have recently demonstrated that the low-frequency photon spectrum of these structures is divided into groups of longitudinal resonances in accordance with the field distribution in the rectangular cross section of the resonator, which shift as a whole when the cross section geometry changes [23]. A simplification that allows understanding the main principles is based on the division into Fabry–Pérot-like resonances along the horizontal and vertical axes of the resonators.

To avoid confusion, we introduce two main definitions that we will use further in the text: *‘galleries’* – individual sets of equidistant longitudinal modes, by analogy with the well-known whispering gallery modes (WGM) of a disk resonator; *‘cascades’* of q-BICs – multiple equidistant anti-crossings, formed by two different galleries in the parametric space of geometrical parameters. The latter are the main result of this work.

## 2 Results

### 2.1 Cascades of q-BICs: numerical demonstration

In general, the formation of q-BICs can be described with phenomenological Friedrich–Wintgen’s Hamiltonian for two resonances (or eigenmodes), which couple to the same radiation channel [19], [24]:

(1) $$H=\left(\begin{array}{cc} \omega_{1} & \kappa \\ \kappa & \omega_{2} \end{array}\right)-i\left(\begin{array}{cc} \gamma_{1} & \sqrt{\gamma_{1}\gamma_{2}}\\ \sqrt{\gamma_{1}\gamma_{2}} & \gamma_{2} \end{array}\right)$$

where *ω*_1,2_ and *γ*_1,2_ are resonant frequencies and half-widths of uncoupled resonant states, respectively; *κ* is the near-field intrinsic coupling between the states, which operates at the same radiation channel; the term $\sqrt{\gamma_{1}\gamma_{2}}$ represents the far-field interference. According to the model (1), the q-BIC regime occurs when the coupling coefficient *κ* has the following form (see Supplementary materials, Section A):

(2) $$\kappa_{q-BIC}=\frac{\left(\omega_{1}-\omega_{2}\right)\sqrt{\gamma_{1}\gamma_{2}}}{\gamma_{1}-\gamma_{2}}$$

Physically, Eq. (2) shows that the strong-coupling regime [17], [18] (the other name for q-BIC) occurs when the difference in radiation losses $\left(\gamma_{1}-\gamma_{2}\right)$ is suppressed, i.e. eigenmodes destructively interfere in the far-field. The effective radiation suppression requires the same symmetry group of eigenmodes [25], which hybridize at the strong-coupling regime and form symmetric and anti-symmetric states of (1) [26]. The latter is a high-*Q* state, which disappears in the far-field on one of the branches in an avoiding crossing.

Notably, the simplest examples of equidistant eigenmodes, which have the same symmetry, were not considered to form the q-BIC set, specifically: eigenmodes of ring and Fabry–Pérot-like modes of cuboids.

**Figure 1:**
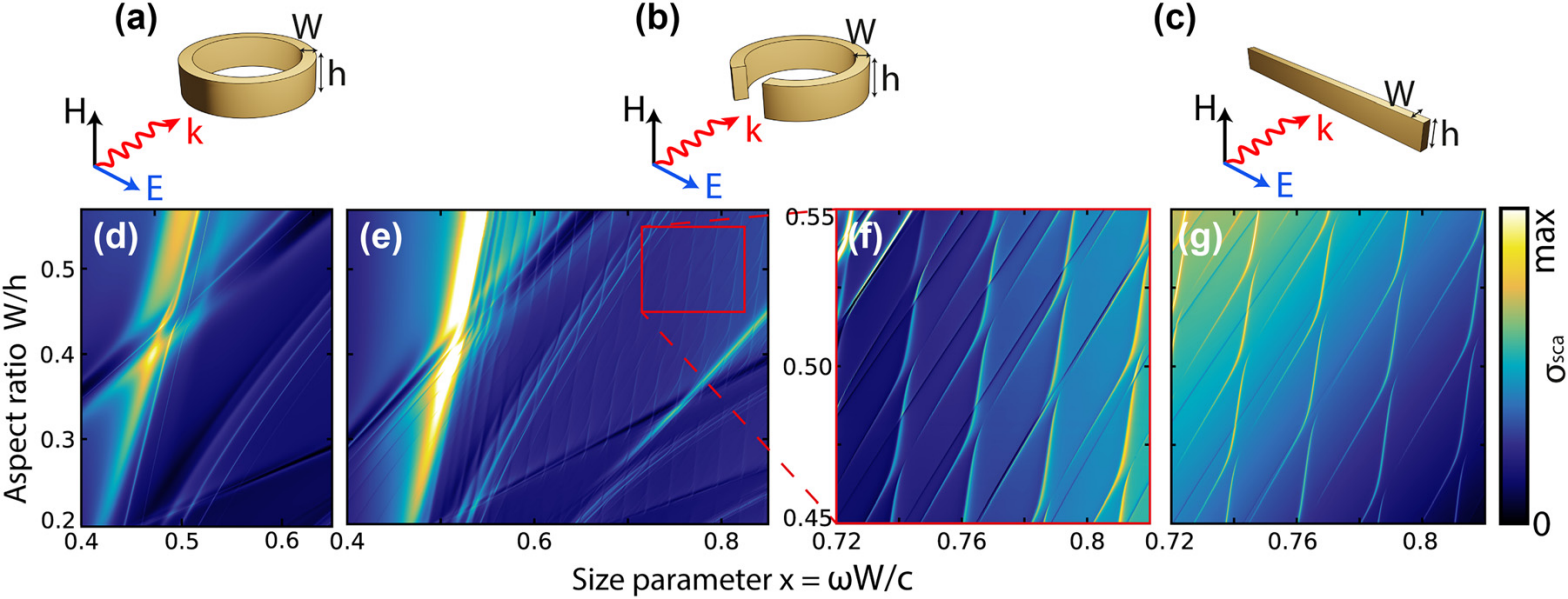
Dielectric resonators supporting cascades of q-BICs and corresponding scattering maps. Top: illustrative models of the structures under study and the plane wave incidence geometry. Bottom scattering maps *σ*_sca_ for ring (d) and 20-degree split ring resonator (e); (f), scaled area of scattering map highlighted in (e) with a red rectangle; (g), the same area as in (f) but for a cuboid. *ɛ* = 43. Size parameter *x* = *ωW*/*c*.

Here we numerically studied the resonant properties of a ring, a split ring and a cuboid, Figure 1. The calculations were performed with the frequency domain solver of COMSOL Multiphysics (for details see Supplementary Materials, section C) [27]. As we demonstrated earlier [28], with a change in only one geometric size of the cross section, all frequencies of galleries of one type will change, but those of the other will mostly not; therefore, the galleries in the spectra will “overlap”, and photonic branches can intersect or demonstrate avoided crossing, depending on the symmetry of the photonic modes. In this case, if the avoided crossing arises, entire sequences of equidistant high-*Q* q-BICs should appear in the scattering spectra. In this paper, we report just such an impressive picture, observed both in experiment and in calculations, and call these equidistant sequences cascades of q-BICs.

An amazing picture of the interference of photonic galleries using the example of three dielectric structures with a rectangular cross section is presented in Figure 1. The figure shows the resonators studied in this work: a dielectric ring, Figure 1a, a split ring, Figure 1b, and a cuboid, Figure 1c, and their corresponding scattering maps *σ*_sca_, which demonstrate cascades of q-BICs in the interference regions of horizontal and vertical galleries. Each scattering intensity map consists of many individual numerically calculated spectra *σ*_sca_ (see Supplementary Materials, Section C); particularly, the maps in Figure 1d and e consist of 601 spectra, and the maps in Figure 1f and g consist of 281 spectra. Each gallery begins with a transverse Fabry–Pérot-like resonance, which is characterized by horizontal (*r*) and vertical (*z*) indices, and continues with equidistant longitudinal resonances with increasing index *m*. In the case of a ring resonator, the quality factor *Q* of the modes of each gallery increases exponentially with increasing *m*.

**Figure 2:**
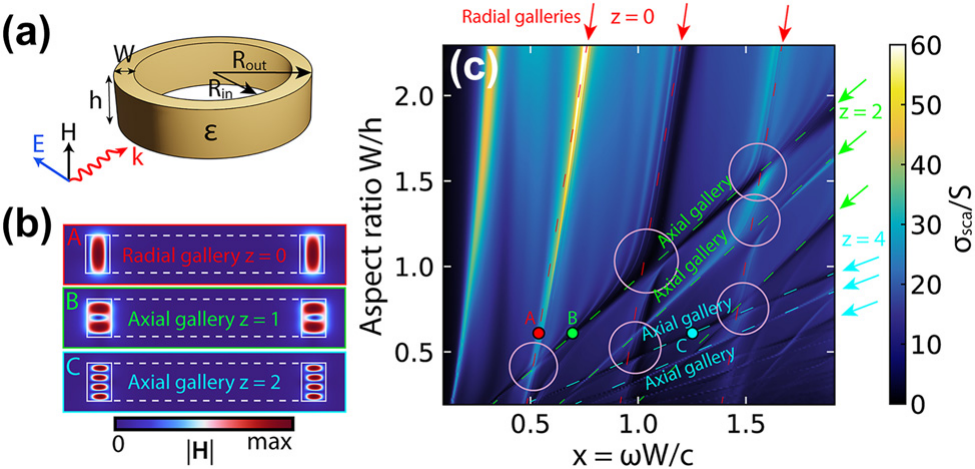
Eigenmodes and cascades of q-BICs of dielectric ring resonators. (a) Narrow dielectric ring and its parameters. (b) The distribution of the magnetic field |**H**| corresponds to the radial (A) and two axial galleries with *z* = 2 (B) and *z* = 4 (C), which are highlighted in (c) with red, green, and blue arrows, circles, and dotted lines. (c) Scattering map *σ*_sca_ of the ring resonator with variable height *h* and constant *R*_in_ and *R*_out_ and, accordingly, constant *W*. The scattering cross section is normalized to the geometric shadow *S* = 2*R*_out_ *h*. Size parameter *x* = *ωW*/*c*. The surrounding space is air. Pink circles mark some of the many avoided crossing areas of the ring galleries. *R*_in_/*R*_out_ = 0.81, *ɛ* = 43.

In a ring resonator, the horizontal and vertical galleries, which due to azimuthal symmetry were called radial and axial, respectively, differ significantly in the distribution of the field in the cross section of the ring resonator, Figure 2b. In the case of TE polarization shown in Figure 2a, radial galleries have a significantly dominant axial component of the magnetic field |*H*_*z*_|, and in the case of axial galleries, the radial component of the magnetic field |*H*_*r*_| is dominant. This division is valid for a wide range of dielectric constants, including conventional refractive indices in the optical range (*ɛ* ≈ 10). The transformation of a dielectric ring into a split ring breaks the axial symmetry, but even in this case, as in the case of a cuboid, separation into galleries in the frequency domain is clearly observed.

It is worth noting that each index in our notation corresponds to the total number of half-waves that fit into the corresponding resonator’s dimension. Thus, for index *m* in the ring, it corresponds to the length of the midline of the ring, and for the split ring and cuboid, the length of the structure, as well as for *r* and *z* in all three structures along the width and height, respectively. For example, in the first radial gallery of the ring resonator there is one maximum of the magnetic field amplitude |**H**| along the radius, Figure 2b, which corresponds to half the wavelength and the radial index *r* = 1; in the second gallery, there are two maximums, this is a whole wavelength, *r* = 2.

It is quite expected that a change in only one of the transverse dimensions of the structure, that is, the width *W* or height *h*, leads to a significant change in frequencies for one transverse Fabry–Pérot-like resonance and has little effect on the other. The surprise is that when one of the transverse dimensions changes, the entire gallery of longitudinal modes shifts, along with “its” Fabry–Pérot-like resonance. For example, a change in *h* at a fixed *W* has little effect on the frequency position of the radial galleries of ring resonator, but leads to a significant change in the equidistant frequencies of the longitudinal modes belonging to the axial galleries, Figure 2c. Note that with increasing axial index *z*, the frequency shift rate of the galleries increases, as can be seen when comparing axial galleries with *z* = 1 and *z* = 2, Figure 2c.

Now we can formulate the key result of the work: when changing the height *h* or width *W* of resonators with a rectangular cross section, regions appear in the scattering spectra in which longitudinal equidistant modes belonging to the horizontal and vertical galleries, depending on the symmetry, demonstrate two different regimes: trivial crossing or avoided crossing with the birth of entire cascades of equidistant q-BICs. Each individual q-BIC arises in accordance with the Friedrich–Wintgen model [24] when two non-orthogonal modes are coupled to the same radiation channel, interfere in the near field, and the avoided crossing arises in the parametric space. In Figure 2c, some of the many avoided crossing areas of radial and axial galleries in the ring resonator are highlighted with pink circles.

**Figure 3:**
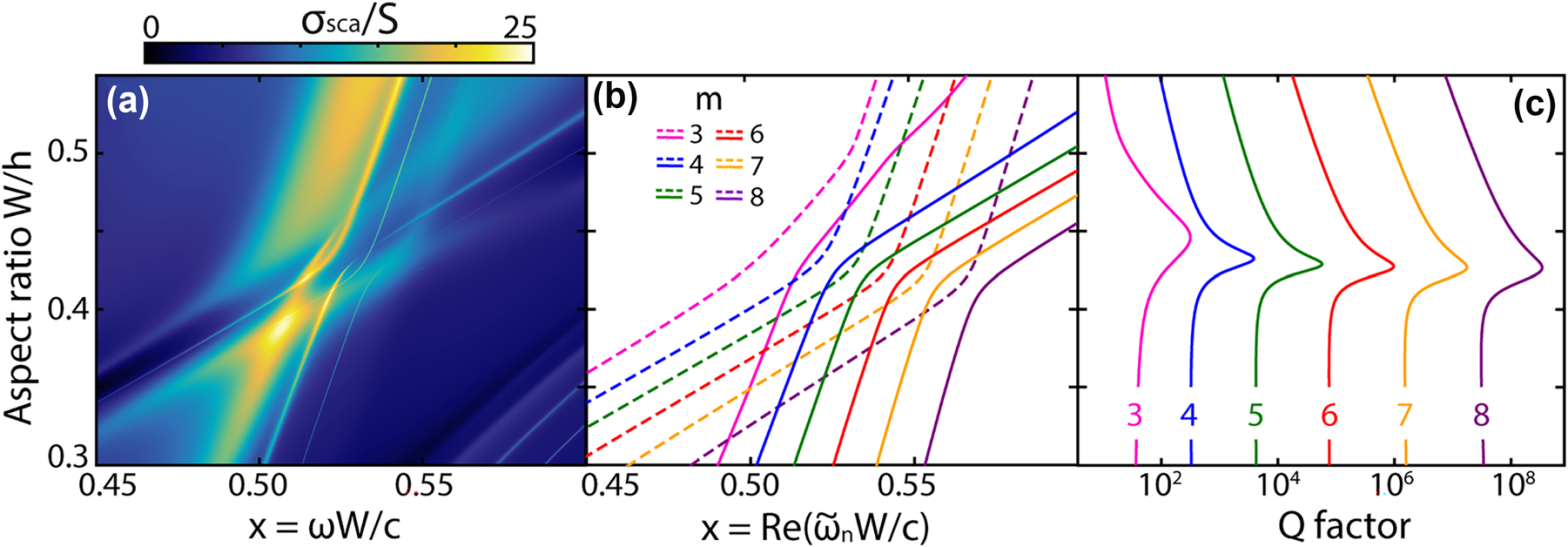
Cascade of q-BICs formed by the radial gallery (*r* = 1, *z* = 0) and axial gallery (*r* = 1, *z* = 2) for dielectric ring resonators without losses. (a) Calculated scattering map *σ*_sca_ and the corresponding eigenvalues: (b) real part of the eigenfrequencies and (c) mode quality factor *Q*. Interfering pairs of modes with the same indices *m* are shown in (b) in the same color. The *Q* factors in (c) correspond to the solid lines in (b). *ɛ* = 43, *R*_in_/*R*_out_ = 0.81, TE-polarized plane wave, size parameter *x* = *ωW*/*c*.

Figure 3 demonstrates in detail the cascade of q-BICs that occurs in the avoided crossing area of the radial (*r* = 1, *z* = 0) and axial (*r* = 1, *z* = 2) galleries observed in the ring resonator, corresponding to the region in the lower left pink circle in Figure 2c. Figure 3a shows a map of the scattering spectra *σ*_*sca*_, and Figure 3b and c demonstrates the eigenvalues ${\tilde{\omega }}_{n}$ for the five highest by *Q* modes with azimuthal harmonics from *m* = 3 to 8. Since each harmonic, in the case of an axially symmetric resonator, constitutes a separate radiation channel, then pairs of modes of two galleries, radial and axial, form avoided crossing and a q-BIC – state in the real part of eigenvalues (see Supplementary Materials, Section B). As already mentioned, in each ring gallery, with increasing index *m*, the *Q* factor of the azimuthal modes increases exponentially [19], and a similar exponential increase in the quality factor is observed for resonances in the q-BIC regime, Figure 3c. Figure 3c demonstrates that all q-BICs are located at approximately the same parameter *W*/*h* ≈ 0.43, which means that there are real dielectric resonators that support cascades of q-BICs.

In the ring with azimuthal symmetry, the scattering channels are separated by azimuthal harmonics *m* which means that only modes of the same azimuthal (*m*) symmetry interact. The split ring does not have azimuthal symmetry, which indicates that all modes belong to the same radiation channel. Consequently, each horizontal gallery mode of the split ring couples to each vertical gallery mode with different coupling coefficients *κ*, Figure 1e (see Supplementary Materials, Section A). The most interesting area on a larger scale is shown in Figure 1f. In the case of a cuboid, there are no significant changes in the behavior of photonic resonances compared to a split ring, but a cuboid resonator is much easier to manufacture for the optical range, which is a significant advantage [29].

Recently, cascades of BICs, with similar distribution in parametric space as that shown in Figure 1g, were observed in [30], [31], for high-contrast gratings upon variation in their thickness. In [32], such cascades were observed for a single waveguide on a substrate by varying the waveguide width. As for q-BIC cascades, to the best of our knowledge, only in [33] this effect was observed experimentally for high-permittivity cylinder in microwave range. Comparison with all these works shows that we achieve a much denser q-BICs distribution in parametric space in the case of ring, split ring or cuboid particles. More importantly, the q-BIC cascades are supported by the single ring resonator with fixed geometry (*W*/*h* ≈ 0.42 in Figure 3c), therefore this effect may be used directly without any changes of the shape, which is not possible for configurations from [30], [31], [32], [33].

## 3 Experimental observation of cascade of q-BICs

**Figure 4:**
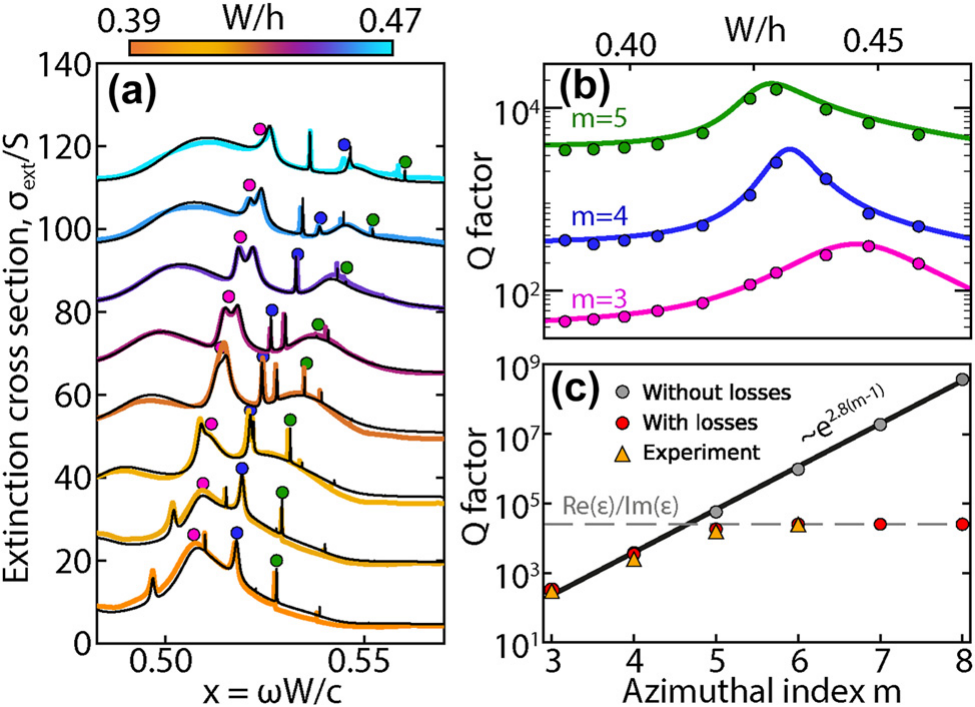
Experimental observation of cascade of q-BICs formed by the radial gallery (*r* = 1, *z* = 0) and axial gallery (*r* = 1, *z* = 2) of ring resonator (a) Comparison of experimental (colored lines) and calculated (black lines) extinction cross section spectra of a ring resonator for various parameters *W*/*h*. The resonances corresponding to the q-BIC are indicated by circles for the three highlighted azimuthal modes *m* = 3 (pink), 4 (blue), and 5 (green). Size parameter *x* = *ωW*/*c*. (b) Mode quality factor Q determined from experimental spectra (circles) and calculated taking into account losses tan(*δ*) ≈ 0.4*e* − 4 (solid lines) depending on the *W*/*h* parameter. (c) Maximum quality factor *Q* of modes with different azimuthal symmetry for the case without losses (gray circles), with losses (red circles), and experimental values (triangles).

The cascade of q-BICs in a dielectric ring with rectangular cross section was experimentally observed from the extinction spectra as the resonator height *h* varied. The experiments were performed with microwave spectroscopy in anechoic chamber (see Supplementary Materials, Section B) [23], [34]. The experimental samples of ring resonator and cuboid were fabricated from ceramic compound (Ca_0.67_La_0.33_) (Al_0.33_Ti_0.67_)O_3_ with high permittivity *ɛ* = 43 and low loss rate tan(*δ*) ≈ 0.4*e* − 4. The main experimental result is presented in Figure 4a, where the experimental spectra of ring resonator are compared with those calculated using quasinormal mode (QNM) theory [35], [36] taking into account 150 modes (see Supplementary Materials, Section C) [37].

The agreement between the calculated and experimental data allows us to judge the correctness of the calculation model and the high quality of the ceramic samples. According to QNM theory, extinction is a linear sum of the contributions of each of the QNM modes $\sigma_{\mathrm{ext}}=\sum_{n}\sigma_{\text{ext}}^{n}+\sigma_{bg}$ , where $\sigma_{\text{ext}}^{n}$ is the Fano contour according to [38]:

(3) $$\sigma_{\text{ext}}^{n}=\sigma_{n}\frac{q_{n}^{2}-1+2q_{n}\Omega_{n}}{\left(q_{n}^{2}+1\right)\left(\Omega_{n}^{2}+1\right)}$$

with the dimensionless frequency Ω_*n*_ = (*ω* − *ω*_*n*_)/*γ*_*n*_, the intensity *σ*_*n*_ and Fano asymmetry parameter *q*_*n*_ of *n*th photonic eigenmode.

To find the intensity parameters *σ*_*n*_ and Fano asymmetry *q*_*n*_, the eigenvalue problem ( ${\tilde{\omega }}_{n}=\omega_{n}-i\gamma_{n}$ , ${\tilde{E}}_{n}$ ) was solved taking into account the resonator losses. The eigenfields ${\tilde{E}}_{n}$ were normalized using the perfectly matched layer (PML) norm [39]. After that, the spectral parameters (*ω*_*n*_, *γ*_*n*_, *σ*_*n*_, *q*_*n*_) for a given incident (background) field **E**_*b*_ were directly calculated using the equations derived in [38]. These theoretically calculated parameters were then used as an initial approximation of the experimental parameters. The contours of narrow azimuthal resonances with indices *m* = 3, 4 and 5, Figure 4a, were approximated by the Fano formula (3) and the values of frequencies and quality factors *Q* were determined. The *Q* values obtained from fitting the spectra were compared with calculated *Q* factors taking into account losses (Figure 4b). The intensity of the harmonic *m* = 6 is limited by material losses in the experimental spectrum, Figure 4c, therefore the quality factor *Q* practically does not increase, but an avoided crossing is observed, and a q-BIC is formed, Figure 3c. Without taking into account losses, the maximum quality factor *Q* of q-BIC grows exponentially with increasing *m* and is approximated by an exponential function. Exponential growth starts from the azimuthal harmonic *m* = 3 and can be approximated as *e* ^2.8(*m*−1)^. In the presence of losses, the maximum quality factor is indicated by a gray dotted line in Figure 4c and corresponds to 1/ tan(*δ*).

**Figure 5:**
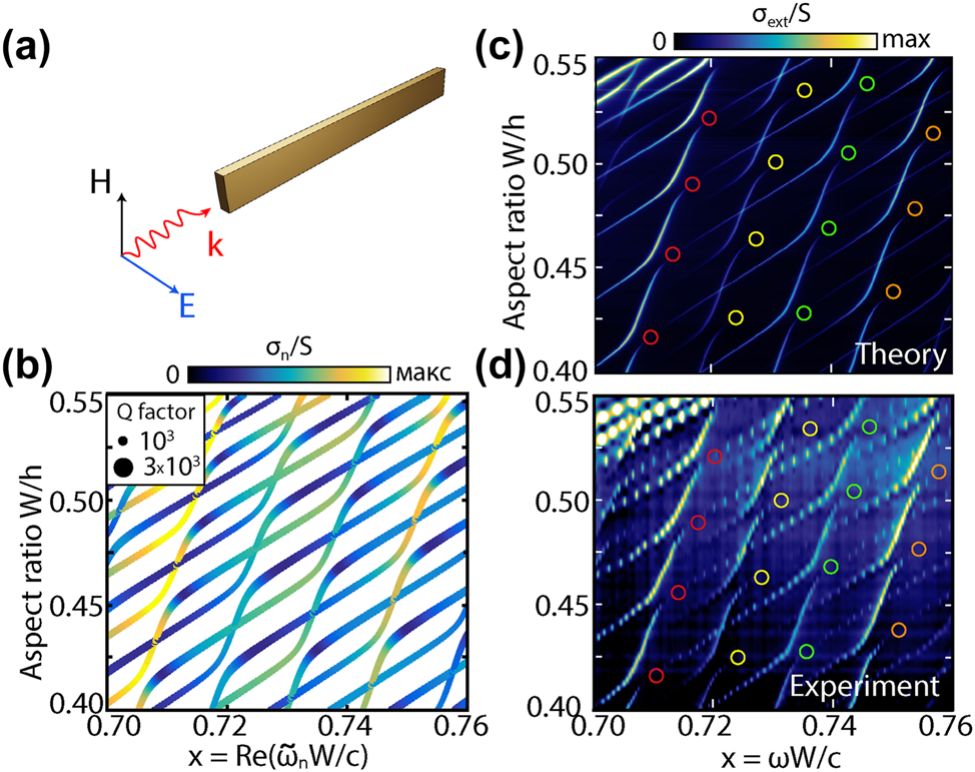
Cascades of q-BICs for the cuboid (a) Schematic representation of a cuboid and an incident wave. (b) Cuboid eigenfrequencies as a function of the *W*/*h* geometry. The colors represent the intensity of each QNM calculated according to [36]. (c) Numerical calculation and (d) experimental maps of extinction *σ*_*ext*_. The resonator length *L* = 108.9 mm, width *W* = 4 mm, permittivity *ɛ* = 43 and losses tan(*δ*) ≈ 0.4*e* − 4.

According to Figure 1g, in the case of a cuboid, the same QNM symmetry leads to multiple anti-crossings between all resonances in the space of the geometric parameter *W*/*h* and frequency. For the experimental demonstration, we used a cuboid with length *L* = 108.9 mm, width *W* = 4 mm, and initial height *h* = 11 mm, Figure 5a. In the selected range of parameters, two extinction maps were obtained: numerically calculated taking into account 350 QNM, Figure 5c, and experimental one, Figure 5d. In experiments, the height was reduced to 7 mm with a step of approximately 0.05 mm. The orientation of the incident field is shown in Figure 5a.

Calculations of *σ*_*n*_ show that in the region of anti-crossing of a pair of modes, one of the QNMs simultaneously increases the quality factor *Q* and decreases the intensity of the scattered field, Figure 5b. Thus, the mode is localized inside the resonator and a large value of the field ${\tilde{E}}_{n}$ is observed. In the optical range, such a resonator can be made of AlGaAs and higher harmonics can be generated [40], [41], [42], which in the parametric space will represent a q-BIC cascade. Note that in the absence of axial symmetry, some resonances may be switched off due to the symmetry prohibition of mode excitation (see Supplementary Materials, Section C).

All presented results can be transferred to the optical frequency range with the difference only in the slope angle of the exponential growth of the quality factor depending on the azimuthal index *m* of the modes in the ring, which is defined by permittivity of the resonator. An example of the implementation of a cascade of q-BICs in a silicon ring resonator can be found in the Supplementary Materials, Section D.

## 4 Cascades of q-BICs as a new platform for machine learning

**Figure 6:**
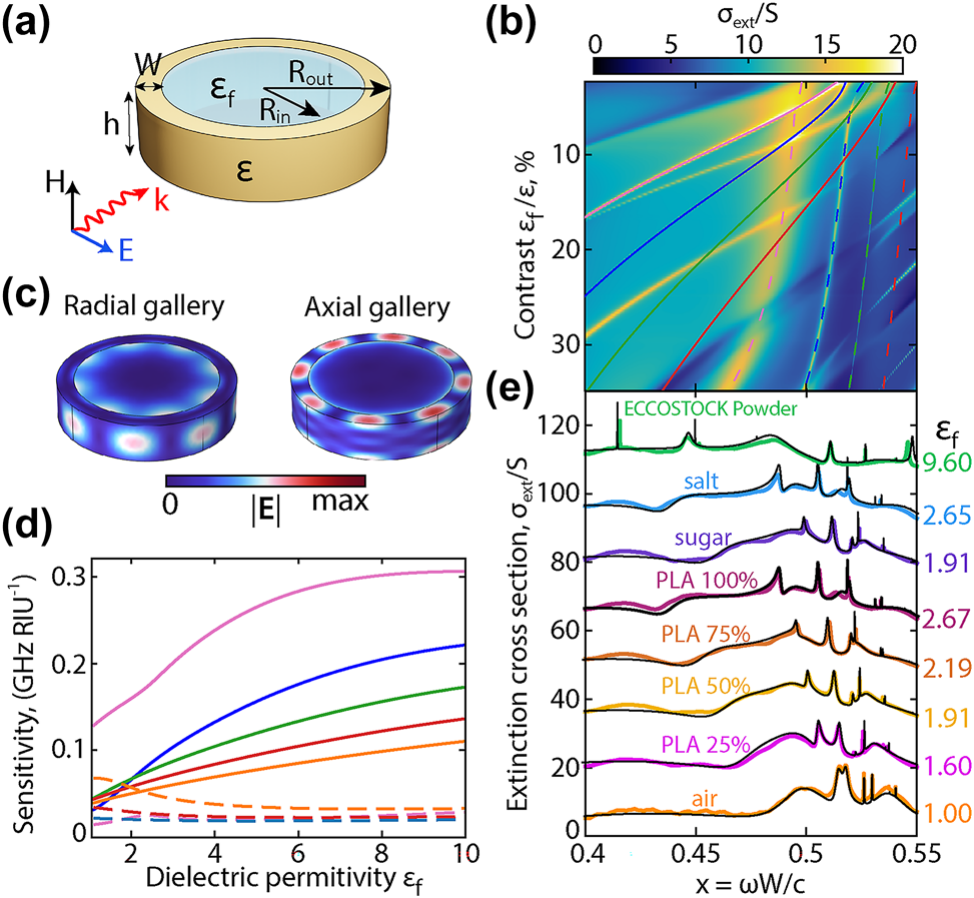
Determination of permittivity using machine learning. (a) Schematic ring parameters and scattering geometry of the sensor. (b) Extinction map of a ring resonator with a filled internal hole as a function of permittivity. Solid and dotted lines indicate the azimuthal harmonics *m* of the radial and axial gallery, respectively. (c) Field distribution for radial and axial modes with azimuthal index *m* = 4 for the filler permittivity *ɛ*_*f*_ = 5. (d) Sensitivity of resonant modes upon changes of the permittivity *ɛ*_*f*_/*ɛ*. (e) Comparison of experimental and calculated extinction spectra taking into account the permittivity determined using machine learning. Size parameter *x* = *ωW*/*c*.

Due to new calculation methods and modern computer devices, artificial intelligence has been incredibly actively used in recent years for solutions in various fields of science [43], [44]. Here we demonstrate that a dielectric ring resonator supporting a cascade of q-BICs, together with machine learning, can be used as a sensor of refractive index for a material located inside the ring resonator hole, Figure 6a. Such sensors have a wide range of different applications, from chemistry and biology to the food industry [45], [46]. A map of extinction spectra was calculated for a wide range of filler-ring contrast *ɛ*_*f*_/*ɛ*, Figure 6b. The spectra were calculated with steps of Δ*ɛ*_*f*_ = 0.5.

The map shows that the change in the permittivity has different impacts on the radial and axial galleries in the ring. Radial ring galleries have a significant shift, while axial ones practically do not change their frequency. The bigger spectral shift of the radial modes is caused by the stronger field penetration inside the filler volume, in contrast to axial modes, which are mostly localized in the ring resonator, Figure 6c. Therefore, the sensitivity of radial gallery modes is higher than that of axial ones, Figure 6d. The relative shift of galleries can be used to determine the possibly-dispersive permittivity of a filler, since this approach differs from a single resonance response and provides a transformation of the group of resonances. However, here we will demonstrate only a simplified version of the method for determining the average real-valued permittivity.

We used the numerically calculated extinction spectra for different filler permittivity as the training data for the neural network (NN), Figure 6b. In our model, the experimental spectra were used for input to the two-layer NN. After data processing, the model provides an average filler permittivity *ɛ*_*f*_ in the range from 1 to 20 (for details see Supplementary Materials, Section E).

The calculated spectra with the permittivity predicted by NN converge with the experimental spectra in all cases, Figure 6e. For PLA plastic with a filling of 25, 50, 75, and 100 %, the values *ɛ*_*f*_ = 1.6, 1.91, 2.19, and 2.67 were obtained, respectively. And for sugar, salt, and ECCOSTOCK powder, the permittivity was determined as *ɛ*_*f*_ = 1.91, 2.65, and 9.6, respectively. In this example, the model allows one to determine the real part of the permittivity with an accuracy of about ∼5% . The model can be adapted for dispersive permittivity measurements by splitting the experimental spectrum into several training intervals, which cannot be provided by single-resonance devices.

A brief comparison with other methods for obtaining permittivity, including methods using a coaxial conductor [47], a waveguide [48], capacitor plates [49], and free-space measurements [50], as well as details of the created NN, are described in Supplementary Materials, Section E.

## 5 Discussion

We demonstrate that key objects of modern photonics – a dielectric ring, a split ring resonator and a cuboid, all with a rectangular cross-section, form q-BIC cascades by the interaction of two galleries of longitudinal resonances; these gallaries formed by transverse Fabry–Pérot-like modes. We show that for a ring resonator the quality factor of each subsequent q-BIC grows exponentially, and when the resonator symmetry is broken, for the case of a split ring resonator and a cuboid, a q-BIC cascade is observed in the parameter space (*ωW*/*c*, *W*/*h*). We confirm our extensive calculations with an experiment performed in the microwave range for both the ring resonator and the cuboid. Finally, we demonstrate that q-BIC cascades provide an excellent platform for multifrequency sensing using machine learning [43]. We used the extinction spectra map as a function of the permittivity of the filled material as training spectra and determined the permittivity of the material placed inside the ring resonator. Cascades of q-BICs enable a new approach for multichannel filtering [51], nonlinear nanooptics [40], resonant optical amplifiers and antennas [18]. For instance, with minimal material losses, like in Si in a transparency window or AlGaAs, the cascades of q-BICs enhance the interaction of light with matter, allowing for efficient generation of higher harmonics [40], [41], [42]. To summarize, we believe that Fabry–Pérot-tronics has the potential to revolutionize fundamental and applied photonics by creating a new platform for multichannel information processing [51].

## Supplementary Material

Supplementary Material Details

## References

[1] Won R. (2019). Into the ‘Mie-tronic’ era. *Nat. Photonics*.

[2] Kivshar Y. (2022). The Rise of Mie-tronics. *Nano Lett.*.

[3] Kuznetsov A. I., Miroshnichenko A. E., Fu Y. H., Zhang J. B., Luk’yanchuk B. (2012). Magnetic light. *Sci. Rep.*.

[4] Evlyukhin A. B. (2012). Demonstration of magnetic dipole resonances of dielectric nanospheres in the visible region. *Nano Lett.*.

[5] Ginn J. C. (2012). Realizing optical magnetism from dielectric metamaterials. *Phys. Rev. Lett.*.

[6] Tzarouchis D., Sihvola A. (2018). Light scattering by a dielectric sphere: perspectives on the Mie resonances. *Appl. Sci.*.

[7] Mie G. (1908). Contributions to the optics of turbid media, especially colloidal metal solutions. *Ann. Phys.*.

[8] Lam C., Leung P., Young K. (1992). Explicit asymptotic formulas for the positions, widths, and strengths of resonances in Mie scattering. *J. Opt. Soc. Am. B*.

[9] Bohren C. F., Huffman D. R. (1986). *Absorption and Scattering of Light by Small Particles*.

[10] Richtmyer R. D. (1939). Dielectric resonators. *J. Appl. Phys.*.

[11] Kerker M., Wang D.-S., Giles C. L. (1983). Electromagnetic scattering by magnetic spheres. *J. Opt. Soc. Am.*.

[12] Limonov M. F., Rybin M. V., Poddubny A. N., Kivshar Y. S. (2017). Fano resonances in photonics. *Nat. Photon.*.

[13] Limonov M. F. (2021). Fano resonance for applications. *Adv. Opt. Photon.*.

[14] Zel’dovich I. B. (1958). Electromagnetic interaction with parity violation. *J. Exp. Theor. Phys.*.

[15] Miroshnichenko A. E. (2015). Nonradiating anapole modes in dielectric nanoparticles. *Nat. Commun.*.

[16] Hsu C. W., Zhen B., Stone A. D., Joannopoulos J. D., Soljačić M. (2016). Bound states in the continuum. *Nat. Rev. Mat.*.

[17] Solodovchenko N., Samusev K., Bochek D., Limonov M. (2021). Bound states in the continuum in strong-coupling and weak-coupling regimes under the cylinder – ring transition. *Nanophotonics*.

[18] Bogdanov A. A. (2019). Bound states in the continuum and Fano resonances in the strong mode coupling regime. *Adv. Photon.*.

[19] Solodovchenko N., Samusev K., Limonov M. (2024). Quadruplets of exceptional points and bound states in the continuum in dielectric rings. *Phys. Rev. B*.

[20] Cao H., Wiersig J. (2015). Dielectric microcavities: model systems for wave chaos and non-Hermitian physics. *Rev. Mod. Phys.*.

[21] Zhou H. (2018). Observation of bulk Fermi arc and polarization half charge from paired exceptional points. *Science*.

[22] Zhang F. (2025). Non-Hermitian singularities in scattering spectra of Mie resonators. *Sci. Adv.*.

[23] Solodovchenko N. (2022). Cascades of Fano resonances in light scattering by dielectric particles. *Mater. Today*.

[24] Friedrich H., Wintgen D. (1985). Interfering resonances and bound states in the continuum. *Phys. Rev. A*.

[25] Gladyshev S., Frizyuk K., Bogdanov A. (2020). Symmetry analysis and multipole classification of eigenmodes in electromagnetic resonators for engineering their optical properties. *Phys. Rev. B*.

[26] Vial B., Hao Y. (2016). A coupling model for quasi-normal modes of photonic resonators. *J. Opt.*.

[27] Gladyshev S. (2024). Fast simulation of light scattering and harmonic generation in axially symmetric structures in COMSOL. *ACS Photon.*.

[28] Chetverikova A. P., Limonov M. F., Sidorenko M. S., Samusev K. B., Solodovchenko N. S. (2023). Radial and axial photonic galleries of dielectric rings. *PNFA*.

[29] Bochkarev M., Solodovchenko N., Samusev K., Limonov M. (2024). Topology and curvature effects in the photonics of ring – split ring – cuboid transitions. *Mater. Today*.

[30] Chang-Hasnain C. J., Yang W. (2012). High-contrast gratings for integrated optoelectronics. *Adv. Opt. Photon.*.

[31] Blanchard C., Hugonin J.-P., Sauvan C. (2016). Fano resonances in photonic crystal slabs near optical bound states in the continuum. *Phys. Rev. B*.

[32] Bezus E. A., Bykov D. A., Doskolovich L. L. (2018). Bound states in the continuum and high-Q resonances supported by a dielectric ridge on a slab waveguide. *Photon. Res.*.

[33] Odit M., Koshelev K., Gladyshev S., Ladutenko K., Kivshar Y., Bogdanov A. (2021). Observation of supercavity modes in subwavelength dielectric resonators. *Adv. Mater.*.

[34] Larsson C., Gustafsson M. (2013). Wideband measurements of the forward RCS and the extinction cross section. *ACES J.*.

[35] Yan W., Faggiani R., Lalanne P. (2018). Rigorous modal analysis of plasmonic resonances. *Phys. Rev. B*.

[36] Lalanne P., Yan W., Vynck K., Sauvan C., Hugonin J.-P. (2018). Light interaction with photonic and plasmonic resonances. *Laser Photon. Rev.*.

[37] Wu T., Arrivault D., Yan W., Lalanne P. (2023). Modal analysis of electromagnetic resonators: user guide for the MAN program. *Comput. Phys. Commun.*.

[38] Bochkarev M., Solodovchenko N., Samusev K., Limonov M., Wu T., Lalanne P. (2024). Quasinormal mode as a foundational framework for all electromagnetic Fano resonances. *arXiv: 2412.11099,*.

[39] Sauvan C., Wu T., Zarouf R., Muljarov E. A., Lalanne P. (2022). Normalization, orthogonality, and completeness of quasinormal modes of open systems: the case of electromagnetism. *Opt. Express*.

[40] Koshelev K. (2020). Subwavelength dielectric resonators for nonlinear nanophotonics. *Science*.

[41] Gigli C., Wu T., Marino G., Borne A., Leo G., Lalanne P. (2020). Quasinormal-mode non-hermitian modeling and design in nonlinear nano-optics. *ACS Photonics*.

[42] Kodigala A., Lepetit T., Gu Q., Bahari B., Fainman Y., Kanté B. (2017). Lasing action from photonic bound states in continuum. *Nature*.

[43] Eisenstein M. (2024). Seven technologies to watch in 2024. *Nature*.

[44] Amarjeet M. (2023). Applications of artificial intelligence in physics. *IJEMMASSS*.

[45] Fan X., White I. M., Shopova S. I., Zhu H., Suter J. D., Sun Y. (2008). Sensitive optical biosensors for unlabeled targets: A review. *Anal. Chim. Acta*.

[46] Parvin T., Ahmed K., Alatwi A. M., Rashed A. N. Z. (2021). Differential optical absorption spectroscopy-based refractive index sensor for cancer cell detection. *Opt. Rev.*.

[47] Blackham D. V., Pollard R. D. (1997). An improved technique for permittivity measurements using a coaxial probe. *IEEE Trans. Instrum. Meas.*.

[48] Chiu T. (2003). Dielectric constant measurement technique for a dielectric strip using a rectangular waveguide. *IEEE Trans. Instrum. Meas.*.

[49] Murata K., Hanawa A., Nozaki R. (2005). Broadband complex permittivity measurement techniques of materials with thin configuration at microwave frequencies. *J. Appl. Phys.*.

[50] Rolfes I., Schiek B. (2004). Calibration methods for microwave free space measurements. *Adv. Radio Sci.*.

[51] Kahn J., Miller D. (2017). Communications expands its space. *Nat. Photon.*.

