# Supplementary material for: Cascades of quasi-bound states in the continuum
Source: Nanophotonics. 2025 Aug 5;14(18):3043–51. doi: 10.1515/nanoph-2025-0238 (PMC12442363; doi:10.1515/nanoph-2025-0238)
Supplement: Supplementary file 1 — Supplementary Material Details [file j_nanoph-2025-0238_suppl_001.pdf]

Supplementary Materials

Nikolay Solodovchenko, Mikhail Bochkarev, Kirill Samusev, and Mikhail Limonov*

Cascades of quasi-bound states in the continuum

Section A. Phenomenological theory of q-BIC

To describe the occurrence of q-BIC cascades, we used Friedrich-Wintgen mechanism, in which radiation losses can be coherently suppressed when there are two interfering modes of close frequency with only one open radiation channel [S1]. In a ring resonator, due to axial symmetry, the radiation channels are separated by the azimuthal index *m*, since the overlap integral of fields **E** of different symmetries in an axially symmetric structure always vanishes. In this case, each mode of the radial gallery interacts with the mode of the axial ring gallery, the amplitudes of which are described by the dynamic equation [S2]:

$$H = \begin{pmatrix} \omega_1 & \varkappa \\ \varkappa & \omega_2 \end{pmatrix} - i \begin{pmatrix} \gamma_1 & \sqrt{\gamma_1\gamma_2} \\ \sqrt{\gamma_1\gamma_2} & \gamma_2 \end{pmatrix}, \tag{1}$$

the frequencies ω_1 and ω_2 and the corresponding damping rates $\gamma_{1,2}$ are the eigenvalues of non-interacting modes. The off-diagonal elements of the Hamiltonian have the meaning of the coupling coefficient in the near field $\varkappa$, and $\sqrt{\gamma_1\gamma_2}$ is the interference in the far field. It is important to note, that it is sufficient to take into account only two modes for a qualitative explanation of the occurrence of a q-BIC, since the eigenfrequencies of other modes operating in the same radiation channel are far from each other.

The characteristic equation for the Hamiltonian (1) is

$$\tilde{\omega}^2 - [(\omega_1 + \omega_2) + i(\gamma_1 + \gamma_2)]\tilde{\omega} + [(\omega_1\omega_2 - \varkappa^2) + i(\gamma_1\omega_2 + \gamma_2\omega_1 - 2\varkappa\sqrt{\gamma_1\gamma_2})] = 0, \tag{2}$$

Now we introduce new notations for simplicity: $\omega_0 \equiv (\omega_1 + \omega_2)/2$; $\Omega \equiv \omega_1 - \omega_2$; $\gamma_s \equiv \gamma_1 + \gamma_2$; $\gamma_d \equiv \gamma_1 - \gamma_2$; $\gamma_0 \equiv \sqrt{\gamma_1\gamma_2}$. Then, Eq. (2) can be rewritten in the next form

$$\left[\tilde{\omega} - \left(\omega_0 + i\frac{\gamma_s}{2}\right)\right]^2 = \frac{1}{4}[(\Omega^2 + 4\varkappa^2 - \gamma_s^2) + 2i(\Omega\gamma_d + 4\varkappa\gamma_0)]. \tag{3}$$

The solution for Eq. (3) is:

$$\tilde{\omega} = \left(\omega_0 + i\frac{\gamma_s}{2}\right) + \frac{1}{2}\sqrt{(\Omega^2 + 4\varkappa^2 - \gamma_s^2) + 2i(\Omega\gamma_d + 4\varkappa\gamma_0)}. \tag{4}$$

Let’s consider this root (Eq. (4)) in detail. We can introduce by definition two quantities: $a \equiv \Omega^2 + 4\varkappa^2 - \gamma_s^2$ and $b \equiv 2(\Omega\gamma_d + 4\varkappa\gamma_0)$. From the basic complex calculus, it is straightforward to show that: $r + is = \sqrt{a + ib} \;\Rightarrow\; (r + is)^2 = a + ib \;\Rightarrow a = r^2 - s^2;\; b = 2rs$. The substitution of $s = b/(2r)$ into equation for a leads to a biquadratic equation for r:

$$r^2 - \frac{b^2}{4r^2} - a = 0 \Rightarrow r^4 - ar^2 - \frac{b^2}{4} = 0. \tag{5}$$

Equation (5) has the next solutions for r and s:

N. Solodovchenko, M. Bochkarev, K. Samusev, M. Limonov School of Physics and Engineering, ITMO University, Saint-Petersburg, Russia; **K. Samusev, N. Solodovchenko, M. Limonov:** Ioffe Institute, Saint-Petersburg, Russia;

$$r_\pm = \pm\frac{1}{\sqrt{2}}\sqrt{a+\sqrt{a^2+b^2}} \quad ; \quad s_\pm = \frac{b}{2r_\pm}. \tag{6}$$

Note that $r_\pm \in \mathbb{R}$ and $r_\pm^2 \geq 0$. Substitution of Eq. (6) in Eq. (4) gives two eigenfrequencies:

$$\widetilde{\omega}_\pm = (\omega_0 + r_\pm) + i\frac{1}{2}\left(\gamma_s + \frac{b}{2r_\pm}\right) =$$

$$= \left(\omega_0 \pm \frac{1}{2\sqrt{2}}\sqrt{a+\sqrt{a^2+b^2}}\right) + i\frac{1}{2}\left(\gamma_s \pm \frac{\sqrt{2}b}{\sqrt{a+\sqrt{a^2+b^2}}}\right). \tag{7}$$

Consider the imaginary part $\frac{b}{\sqrt{a+\sqrt{a^2+b^2}}}$ as a function of Ω. We can find the roots for the derivative $\partial\left(\frac{b}{\sqrt{a+\sqrt{a^2+b^2}}}\right)/\partial\Omega = 0$, which are: $\Omega_1 = \frac{\varkappa\gamma_d}{\gamma_0}$ and $\Omega_2 = -\frac{\gamma_0\gamma_d}{\varkappa}$. The main term is Ω_1, which provides exact zero for the derivative, therefore the $\widetilde{\omega}$ at Ω_1 always has an extremum value in the imaginary part. Finally, substitution of Ω_1 in Eq. (7) for $\omega_\pm$ correspond the q-BIC with coupling coefficient:

$$\varkappa_{\text{q-BIC}} = \frac{(\omega_1-\omega_2)\sqrt{\gamma_1\gamma_2}}{(\gamma_1-\gamma_2)}. \tag{8}$$

In an ideal case, if this condition (8) is met, one of the solutions to the Hamiltonian becomes purely real, and the second will have double losses. The purely real eigenvalue of the Hamiltonian will correspond to a bound state in the continuum with infinite quality factor. But for a finite open resonator, unlike periodic metasurfaces [S2], an infinite quality factor cannot be realized as a consequence of finiteness in all three dimensions, but if this condition is met, the quality factor of one of the modes increases by several orders of magnitude.

The coupling coefficient $\varkappa$ of two modes can be obtained by fitting the eigenvalues of each azimuthal harmonic m using the solution of Hamiltonian (1). Using this method, the eigenvalues were fitted and the coupling coefficients $\varkappa$ for each harmonic were obtained, Figure A1. When the $\varkappa$ is close to be real valued, the strong coupling regime is achived.

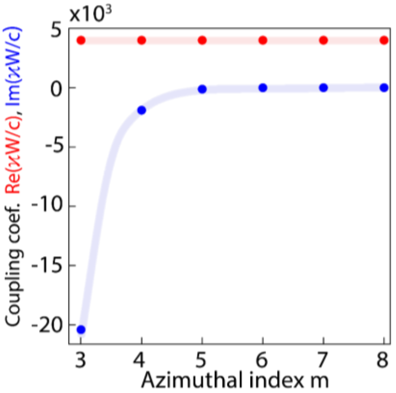

Fig. A1 | Coupling coefficient $\varkappa$ for modes with azimuthal indices m = 3-8. The red and blue dots represent the real and imaginary parts of the coupling coefficient, respectively.

Section B. Experimental methods

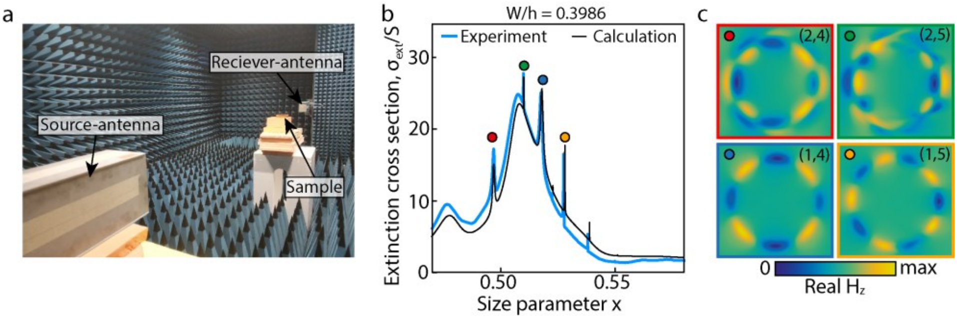

Fig. B1 | **(a)** The photograph of the experimental setup (anechoic chamber). **(b)** Calculated and experimental extinction spectra σ_{ext} and **(c)** calculated near-field distributions. The ring resonator parameters are given in the caption to Fig. 2 (c)

Experimental near-fields and far-field extinction spectra were measured in an anechoic chamber (Fig. B1(a)). Scattering spectra were measured using broadband rectangular horn antennas (frequency range from 0.75 to 18 GHz) and Vector Network Analyzer "Rohde & Schwarz". The experimental sample was located in the middle of the distance between the source and receiver antennas, the total distance between which was about 4 meters. This experimental setup is suitable for transmission coefficient measurements. If the free-space transmission coefficient S_{21}^{b} and this coefficient with presence of the resonator S_{21} are obtained, then the imaginary part of the relation $S_{21}/S_{21}^{b}-1$ is proportional to the scattering cross-section, according to optical theorem [S3]. The near-field magnetic field distribution was measured using only one radiating horn antenna; the second antenna was replaced by a Langer EMV Technik magnetic field probe attached to an automatic scanner.

Our experiments were carried out on a sample with an outer radius R_{out} = 57.1 mm, an inner radius R_{in} = 46.2 mm, a height h in the range from 23.5 to 27.7 mm, corresponding to W/h from 0.393 to 0.464, which covers the entire required range of geometric parameters. The dielectric constant of a ceramic sample made of $(Ca_{0.67}La_{0.33})(Al_{0.33}Ti_{0.67})O_3$ is $\varepsilon \approx 43$, and the loss is $\tan\delta \approx 0.4e-4$ at 3GHz. Additional details on the samples preparation can be found in [S4]. All experimental data were collected in the frequency range from 2 to 4 GHz and TE polarized wave.

A ring with parameters R_{in}/R_{out} = 0.81 and W/h = 0.429 was used as a sensor to determine the dielectric constant (Fig. 6). The inner hole was filled with PLA plastic with varying degrees of filling (25, 50, 75 and 100%) and lossy substances such as sugar, salt and ECCOSTOCK Powders [S5] the effective dielectric constant of which depends on density.

We used COMSOL Multiphysics to calculate extinction spectra σ_{ext}, eigenvalues, and field distributions. Figure B1 (b,c) demonstrates the possibility of unambiguous interpretation of resonances in the anti-crossing region due to the calculation of the field distribution in the resonator plane. Once again, we note the excellent agreement between the experimental and calculated extinction spectra. The magnetic field distribution in the cross-sectional plane of the ring resonator is shown in Fig. 2c.

Two low-frequency resonances (red and green circles) with two field maxima along the radius belong to the axial gallery (*r*=2, *z*=2, *m*=4,5) (marked in Fig. B1(b) with the green circle), and resonances with one maximum along the radius (blue and orange) belong to the radial gallery (*r*=1, *z*=0, *m*=4,5), marked with red circle. The aspect ratio *W*/*h*=0/3986 determines the position of the resonances immediately after the anti-crossing of the two galleries.

Section C. Numerical calculations

The numerical and theoretical description of photonic properties of dielectric resonators was obtained with COMSOL Multiphysics package, which provide calculations of scattering spectra, eigenvalues, and field distributions. The scattering maps of the dielectric ring were calculated taking into account axial symmetry, which allows to use 2D geometry. To calculate the scattering spectra in the low-frequency range, it is enough to sum the finite series of cylindrical harmonics [S6]. In all calculations, the scattering cross-section σ_{sca} was normalized to the geometric shadow, which is equal to $S = 2R_{out}h$ for a dielectric ring resonator and $S = Lh$ for a cuboid.

Scattering spectra obtained experimentally or numerically were interpreted used the QNM theory [S7, S8], which makes it possible to obtain the scattering response from each mode separately under a plane wave excitation, the sum of all mode contributions provide the total scattering spectra. To reproduce the experimental scattering spectrum in Fig. 4, we account for 150 quasinormal modes, which were normalized with PML-norm [S9].

Using the QNM theory, it can be demonstrated that when changing the incident wave incidence orientation, some of the eigenmodes and q-BIC can be forbidden due to symmetry [S10]. For this purpose, we calculated the intensities of eigenmodes responses considering the incident plane wave orientation for the case of $\varphi = 0^o$ (**k** parallel to the width of the cuboid. Fig. C1, left) and $\varphi = 90^o$ degrees (**k** parallel to the length of the cuboid. Fig. C1, right).

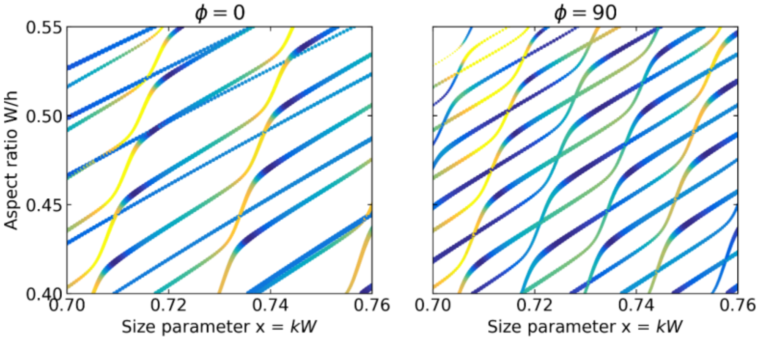

Fig. C1 | Map of eigenvalues for the case of two plane wave incidence orientations: (left, **k** parallel to the width of the cuboid) $\varphi = 0^o$ deg and (right, **k** parallel to the length of the cuboid) 90° deg. Structure: cuboid with the same parameters as in Fig. 5 in the main text.

In the case of split ring resonator and cuboid, both the amplitude and the line shape of the resonance depend on the incidence angle, and the eigenvalues remain constant. For example, Fig. C1 shows that for different orientations of the cuboid relative to the incident plane wave, for the case of 0 degrees of incident wave, it is possible to observe twice as few resonances and anti-crossings in scattering as for 90 degrees.

Section D. Cascades of q-BICs in near infrared region

To demostrate the cascades of q-BICs in resonant nanostructures we consider the ring with permittivity $\varepsilon \approx 12.145 + i6.97 \cdot 10^{-13}$ [S11], which correspond to Silicon at $\lambda = 1.45$ um near the telecom wavelength, and geometrical parameters: radius ratio R_{in}/R_{out} = 0.81, and variable radius-to-height ratio R_{out}/h in the range from 1.4 to 2.6. Indeed, the cascades are observed for the optical-range materials in both eigenfrequenciec and extinction spectra, as shown in Fig. D1. For the azimuthal number $m = 10$ the Q factor achives magnitude of 10^6 (Fig. D1(b)), and the increase of Q factor for neighbour harmonics can be fitted with the exponential function with coefficient of $1.82m$ (Fig. 1D (d)).

When the radius-to-height ratio reaches R_{out}/h ~ 2.13, we obtain nanoresonator, which support the cascade of q-BICs. In this particular case parameters of the ring are the next: width R_{out}-R_{in} = 215 nm; outer radius R_{out} = 1130 nm and height h = 530 nm.

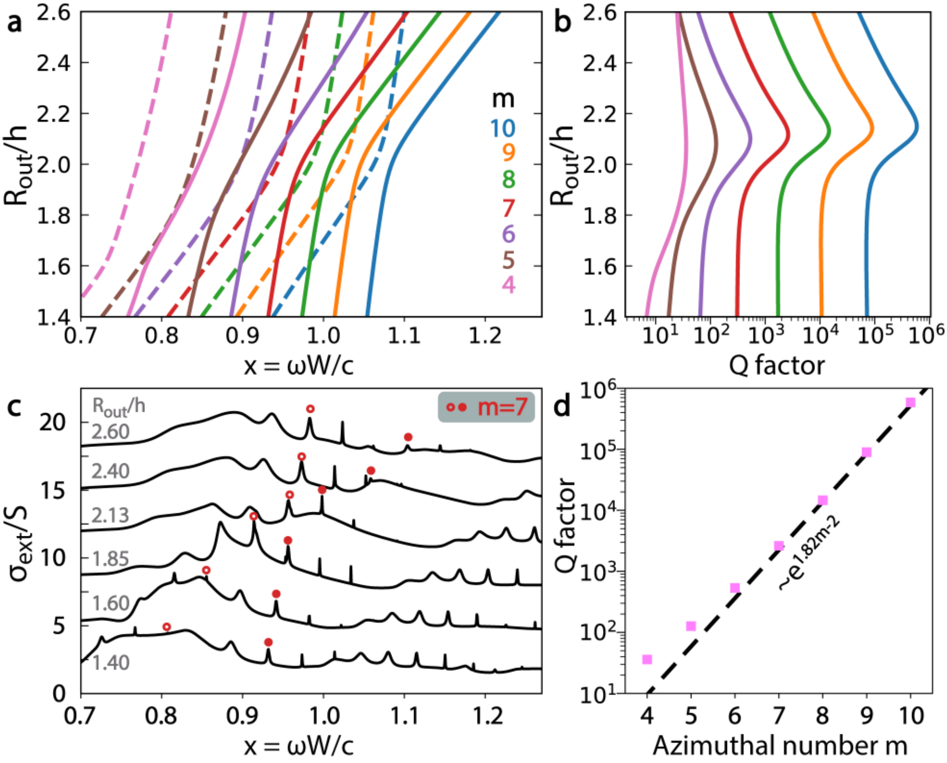

Fig. D1 | Cascades of q-BICs for nanoresonator with permittivity $\varepsilon \approx 12.145 + i6.97 \cdot 10^{-13}$. (a) Formation of anti-crossings for a several azimuthal harmonics from m = 4 to m = 10 (solid-curve branches are the high-Q branches) upon changes in the aspect ratio R_{out}/h. (b) Q factor dependencies on the aspect ratio R_{out}/h for the high-Q branches. (c) Calculated extinction spectra for a several aspects ratios R_{out}/h. (d) Close-to-exponential growth of the Q factor with increase of the azimuthal number m. The black dashed curve correspond to the fit function $e^{1.82m-2}$, the pink markers shows the maximal value of Q factor for branches in (b).

Section E. Neural Network model for determining permittivity from extinction spectra

We used “Python” programming language and “TensorFlow” library to create the model of Neural Network (NN). The scattering map from Fig. 4 (b) in the main text was used as the training data. The model tree is shown in Fig. E1., where the number of input neurons corresponds to the number of frequency points of the calculated spectra (N). The experimental spectrum was fed to the input of the model. The data were then processed by two neuron layers by changing the dimensionality and applying the «ReLu» activation function, and in the last layer, the data dimensionality was reduced to a regular column vector using the «Softmax» activation function. The output of the “Softmax” is a column vector of the probabilities that a given spectrum corresponds to some filler material with permittivity ε_f in the range from 1 to 20. The scalar product of the probability and permittivity vectors is equal to the average value of the permittivity in the frequency range under study.

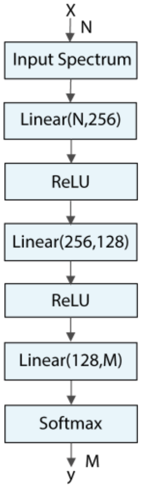

Fig. E1 | The block-scheme of Neural Network with two neural layers with the “ReLu“ activation function, and the additional output layer with “Softmax“ activation function.

There are many methods for determining the permittivity in the microwave range, including methods using a coaxial conductor [S12], a waveguide [S13], capacitor plates [S14], and free-space measurements [S15]. A coaxial conductor is convenient for handling liquids and semi-solid materials. Often, this approach requires the purchase of expensive equipment. All methods are typically used for small samples (several hundreds of micrometers thick) with low losses and provide accuracy ~1–3% [S12, S14]. In this work, we propose an alternative setup of the contactless method for macroscopic samples, in which the filler material is placed inside the hole of ring resonator, and achieve accuracy ~5%, which is sufficient for demonstrating a proof of concept.

As for the optical range, we believe that the cascades of q-BICs can be used in sensing applications and for amplification of the second harmonic generation, however these effects are out of scope of this work.

References to supplementary materials

S1. H. Friedrich and D. Wintgen, "Interfering resonances and bound states in the continuum," Phys. Rev. A, 32, 3231 (1985).

S2. C. W. Hsu, B. Zhen, A. D. Stone, J. D. Joannopoulos, and M. Soljačić, Bound states in the continuum, Nat. Rev. Mat. 1, 1 (2016).

S3. C. Larsson and M. Gustafsson, "Wideband measurements of the forward RCS and the extinction cross section," ACES Journal, vol. 28, no. 12, pp. 1145-1152, 2013.

S4. N. Solodovchenko, M. Sidorenko, T. Seidov, I. Popov, E. Nenasheva, K. Samusev, M. Limonov, "Cascades of Fano resonances in light scattering by dielectric particles," Materials Today, vol. 60, pp. 69-78, 2022.

S5. Properties of ECCOSTOCK, https://www.laird.com/sites/default/files/2018 11/DS%20ECCOSTOCK%20HiK%20Powder.pdf

S6. S. Gladyshev, O. Pashina, A. Proskurin, A. Nikolaeva, Z. Sadrieva, M. Petrov, A. Bogdanov, K. Frizyuk, "Fast simulation of light scattering and harmonic gen eration in axially symmetric structures in COMSOL," ACS Photonics, vol. 11, no. 2, pp. 404-418, 2024.

S7. W. Yan, R. Faggiani, P. Lalanne, "Rigorous modal analysis of plasmonic resonances," Phys. Rev. B, vol. 97, no. 205422, 2018.

S8. P. Lalanne, W. Yan, K. Vynck, C. Sauvan, and J.-P. Hugo nin, "Light Interaction with Photonic and Plasmonic Reso nances," Laser Photonics Rev., vol. 12, np. 5, pp. 1700113, 2018.

S9. C. Sauvan, T. Wu, R. Zarouf, E. A. Muljarov, and P. Lalanne, "Normalization, orthogonality, and completeness of quasinormal modes of open systems: the case of electro magnetism," Opt. Express, vol. 30, no. 5, pp. 6846-6885, 2022.

S10. M Bochkarev, N Solodovchenko, K Samusev, M Limonov, T Wu, P. Lalanne, "Quasinormal mode as a foundational framework for all electromagnetic Fano resonances," 2024, arXiv: 2412.11099.

S11. C. Schinke, P. C. Peest, J. Schmidt, R. Brendel, K. Bothe, M. R. Vogt, I. Kröger, S. Winter, A. Schirmacher, S. Lim, H. T. Nguyen, D. MacDonald. Uncertainty analysis for the coefficient of band-to-band absorption of crystalline silicon. *AIP Advances* **5**, 67168 (2015). Refractive index database "RefractiveIndex.info": https://refractiveindex.info/?shelf=main&book=Si&page=Schinke

S12. D. V. Blackham, and R. D. Pollard, "An Improved Techniquefor Permittivity Measurements Using a Coaxial Probe," IEEE Transactions on Instrumentation and Measurement, vol. 46,no. 5, 1997, https://doi.org/10.1109/APEIE.2018.8546230.

S13. T. Chiu, "Dielectric constant measurement technique for a dielectric strip using a rectangular waveguide," IEEE Transactions on Instrumentation and Measurement, vol. 52, no. 5, pp. 1501 - 1508, 2003, https://doi.org/10.1109/TIM.2003.817904
S14. K. Murata, A. Hanawa, and R. Nozaki "Broadband complex permittivity measurement techniques of materials with thin configuration at microwave frequencies," J. Appl. Phys., vol. 98, no. 084107, 2005, https://doi.org/10.1063/1.2115099.
S15. I. Rolfes, and B. Schiek, "Calibration methods for microwave free space measurements," Adv. Radio Sci., vol. 2, pp. 19- 25, 2004, https://doi.org/10.5194/ars-2-19-2004.
